# Chondrogenic Differentiation of Human Mesenchymal Stem Cells via SOX9 Delivery in Cationic Niosomes

**DOI:** 10.3390/pharmaceutics14112327

**Published:** 2022-10-28

**Authors:** Natalia Carballo-Pedrares, Clara Sanjurjo-Rodriguez, Jose Señarís, Silvia Díaz-Prado, Ana Rey-Rico

**Affiliations:** 1 Centro de Investigacións Científicas Avanzadas (CICA), Universidade da Coruña, As Carballeiras, s/n. Campus de Elviña, 15071 A Coruña, Spain; 2 Institute of Biomedical Research of A Coruña (INIBIC), University Hospital Complex A Coruña (CHUAC), Galician Health Service (SERGAS), 15006 A Coruña, Spain

**Keywords:** niosomes, nioplexes, human mesenchymal stem cells, SOX9, chondrogenesis

## Abstract

Gene transfer to mesenchymal stem cells constitutes a powerful approach to promote their differentiation into the appropriate cartilage phenotype. Although viral vectors represent gold standard vehicles, because of their high efficiency, their use is precluded by important concerns including an elevated immunogenicity and the possibility of insertional mutagenesis. Therefore, the development of new and efficient non-viral vectors is under active investigation. In the present study, we developed new non-viral carriers based on niosomes to promote the effective chondrogenesis of human MSCs. Two different niosome formulations were prepared by varying their composition on non-ionic surfactant, polysorbate 80 solely (P80), or combined with poloxamer 407 (P80PX). The best niosome formulation was proven to transfer a plasmid, encoding for the potent chondrogenic transcription factor SOX9 in hMSC aggregate cultures. Transfection of hMSC aggregates via nioplexes resulted in an increased chondrogenic differentiation with reduced hypertrophy. These results highlight the potential of niosome formulations for gene therapy approaches focused on cartilage repair.

## 1. Introduction

Human mesenchymal stem cells (hMSCs) have been widely investigated in the field of regenerative medicine due to their tri-lineage differentiation capacity and feasibility to induce tissue remodeling [1]. Specifically, hMSCs have gained special attention in the treatment of cartilage and bone lesions, being the recruited population during marrow stimulation techniques [1,2]. However, the number of hMSCs able to differentiate chondrocytes reaching cartilage lesion by this technique is low and age dependent. Hence, its outcome normally led to the formation of fibrocartilage, a tissue with different properties to the hyaline native tissue of the joint [3]. Therefore, the stimulation of hMSCs with chondrogenic factors to elicit a superior therapeutic performance of this outstanding cell population is under active investigation [4].

Gene transfer to hMSCs has emerged as an appealing technology to directly transfer genes encoding for therapeutic factors into this cell population, avoiding common limitations associated with the use of recombinant factors (short half-life, need of repeated doses, side-effects) [5]. Current potential candidates to promote hMSCs chondrogenesis include members from the superfamily of the transforming growth factor beta (TGF-β) [6,7], the bone morphogenetic proteins (BMPs) [8,9], the fibroblast growth factor 2 (FGF-2) [10] or the insulin-like growth factor I (IGF-I) [11], among others. Notwithstanding, the use of these candidates remains restricted by the limited expression of specific matrix components (FGF-2) [12], inadequate chondrogenic differentiation (IGF-I) [13] or hypertrophy (TGF-β, BMPs) [14,15]. In contrast, the sex-determining region Y-type high-mobility group box 9 (SOX9) promotes chondrocytes differentiation by enhancing both aggrecan and type-II collagen gene expression, while restricting terminal differentiation and hypertrophy [16,17,18]. Hereby, SOX9 may be the most suitable factor to refine MSCs chondrogenesis. Of note, most studies involving SOX9 overexpression to promote MSCs chondrogenic differentiation have been performed via viral vectors [19,20,21,22]. Conversely, despite representing the safest tools for gene transfer able to overcome immunogenicity and tumorigenic risks associated with viral vectors, only a few studies have reported the use of non-viral vectors for SOX9 overexpression in hMSCs [21,23,24].

Current gene transfer approaches via non-viral vectors involve the complexation of negatively charged DNA molecules with cationic polymers or lipids to promote their entry in the cell. Among cationic lipids, liposomal vectors have been widely investigated as gene delivery vectors to transfect a variety of cells and tissues [25,26]. However, the high electropositivity required for both DNA complexation and efficient cell internalization normally led to elevated cytotoxicity, considerably precluding the therapeutic potential of these carriers [26,27]. Recently, niosomes have shown to be attractive tools for developing improved gene delivery formulations due to their higher stability and lower toxicity when compared with classical liposomes [28]. These properties have been attributed to the different chemical composition of niosomes from liposomes, by substituting the phospholipids for non-ionic surfactants [29]. Moreover, their low cost and straightforward production make niosomes promising gene delivery systems compared with classical non-viral systems [30].

Based on this, the aim of this study was to develop for the first time new non-viral formulations based on niosomes to promote an effective chondrogenesis of MSCs. As the nanocarriers’ composition may significantly impact their transfection efficiency, being beyond dependent on the cell type and uptake mechanism [31], we tested two different niosome formulations for hMSCs gene transfer. Hence, two niosome formulations were produced using a reverse phase evaporation technique [28] by fixing the composition of the cationic lipid 1,2-di-O-octadecenyl-3-trimethylammonium propane (DOTMA) and the helper lipid squalene and varying the composition of the non-ionic surfactant using polysorbate 80 solely (P80) or P80 combined with poloxamer 407 (P80PX). Resulting nioplexes from the complexation of niosomes with the reporter plasmid *lacZ* (p*lacZ*) were characterized in terms of size, polydispersity index (PDI), charge distribution, capacity of DNA protection, and complexation ability. Further, transfection efficiency, cytotoxic profiles, and cell uptake mechanisms of nioplexes were studied in hMSC primary cultures using the reporter plasmids p*lacZ* and a plasmid encoding for the green fluorescent protein (pGFP). Finally, bioactivity of the most suited formulations was tested by delivering a chondrogenic plasmid encoding for the transcription factor SOX9 (p*sox9*) in hMSC aggregate cultures. Genetic modification of hMSC monolayer cultures via P80PX nioplexes resulted in an increment of both, transfection efficiency and cell viability, compared to those cells transfected with P80 or Lipofectamine complexes, respectively. Moreover, transfection of hMSC aggregates via P80PX/p*sox9* nioplexes resulted in increased chondrogenesis with reduced hypertrophy. These results highlight the potential of P80PX-based niosome formulations for gene therapy approaches focused on cartilage repair.

## 2. Materials and Methods

### 2.1. Materials

All reagents were from Gibco ThermoFisher Scientific (Madrid, Spain) except where otherwise indicated. Polysorbate 80 (Tween 80, 1311.7 g/mol, HLB 15.0) and squalene (MW 410.321 g/mol) were obtained from Alfa Aesar (Kandel, Germany). 1,2-di-O-octadecenyl-3-trimethylammonium propane (chloride salt) (DOTMA, MW 670.575 g/mol) was purchased from Avanti Polar Lipids (Alabaster, AL, USA). Poloxamer 407 (PX; 14,600 g/mol, HLB 18–23) was kindly provided by BASF (Ludwigshafen, Germany).

The 1,9-Dimethyl-Methylene Blue Dye (DMMB), L-cysteine, toluidine blue and recombinant TGF-β3 (TGF-β3) were purchased from Sigma-Aldrich (St. Louis, MI, USA). Eosin Y and Harris hematoxylin solution were purchased from Carl Roth (Karlsruhe, Germany) and chondroitin sulfate C from TRC (Toronto, ON, Canada).

The anti-type II collagen (II-II6B3) antibody was purchased from DSHB (Iowa, IA, USA) and the anti-type X collagen from Sigma Aldrich (St Louis, MO, USA). The anti-SOX9 (E-9) and the anti-type I collagen (COL-1) were purchased from Santa Cruz Biotechnology (Heidelberg, Germany). The biotinylated secondary antibody (Ig G H+L) and the ABC and DAB reagents were obtained from Vector Laboratories (Alexis Deutschland GmbH, Grünberg, Germany).

The PE-conjugated anti-human CD34, FITC-conjugated anti-human CD45, PE-conjugated anti-human CD73, FITC-conjugated anti-human CD90, FITC-conjugated anti-human CD105, FITC and PE isotypes were obtained from BD Biosciences (Madrid, Spain).

The WST-1 and the First Strand cDNA Synthesis Kit for RT-PCR were obtained from Roche (Mannheim, Germany) and the β-glo from Promega (Madison, WI, USA). Label IT^®^ Nucleic Acid Labeling kit Cy3 was purchased from Mirus Bio (Madison, WI, USA). RNeasy Kit was obtained from Qiagen (Hilden, Germany) and Brilliant SYBR Green QPCR Master Mix from Stratagene Agilent Technologies (Waldfronn, Germany).

The p0DB-001_pEGFP-N1 (pGFP; bp 4,733) and pACP-h*sox9* (p*sox9*, bp 6,915) were gifts from Dr. Romero-Saavedra and Prof. M. Cucchiarini, respectively.

### 2.2. Niosome Preparation

Polysorbate 80 solely (15 mg; P80) or combined with poloxamer 407 (7.5 mg P80 and 7.5 mg poloxamer; P80PX), squalene (10.3 mg) and DOTMA (3 mg) were dissolved together in dichloromethane (2 mL) and emulsified with 5 mL of Opti-MEM medium. The mixture was sonified for 30 s at 40% amplitude using a UP200S Sonifier (Hielscher Ultrasound Technology, Teltow, Germany) with a 1 mm probe. The organic solvent was then removed by evaporation under magnetic stirring for 3 h at room temperature, obtaining the dispersion of niosomes in the aqueous medium (600 ng/mL DOTMA final concentration).

### 2.3. Plasmid Propagation and Formation of Nioplexes

The pCMV-SPORT-βgal reporter plasmid (p*lacZ*), pGFP and p*sox9* were propagated, purified and quantified following standard procedures. Labelling of p*lacZ* with Cy3 was performed using a Label IT Nucleic Acid Labeling kit by following the guidelines from the supplier.

Nioplexes were formed by mixing an appropriate volume of a stock solution of p*lacZ*, pGFP or p*sox9* (300 µg/mL; always 700 ng of plasmid) with different volumes of niosomes (P80 or P80PX) in Opti-MEM medium to get cationic lipid/DNA mass ratios (*w/w*) of 2.5/1, 5/1, 10/1, 15/1 and 20/1 or estimated cationic amino groups (N) to nucleic acid anionic phosphate groups (P) ratios of 1, 2, 5, 7 and 10, respectively [28]. The mixtures were allowed to stand for 30 min at room temperature.

### 2.4. Size and Zeta Potential

The hydrodynamic diameter (triplicates), polydispersity index (PDI) (triplicates) and zeta potential (triplicates) of both niosomes and nioplexes were measured in Opti-MEM medium at 25 °C by Dynamic Light Scattering (DLS) and Electrophoretic Light Scattering (ELS) using a NanoBrook 90Plus Zeta (Brookhaven Instruments Corporation, Holtsville, NY, USA).

### 2.5. Agarose Gel Electrophoresis

The capacity of P80 and P80PX niosome formulations to condense DNA was evaluated by agarose gel electrophoresis assay as previously described [28]. Nioplexes were prepared as depicted in Section 2.3, DNA bands were stained with SYBR Green (0.01% in TBE), and images were observed under a digital Chemi-DocTM MP Imaging System (Bio-Rad, Madrid, Spain).

### 2.6. Evaluation of Niosomes Complexation Ability

The ability of niosomes to bind and complex DNA (always 500 ng p*lacZ*) was evaluated by means of a fluorescence-exclusion titration assay [32]. Fluorescence measurements (triplicates) were performed in 96 black well-plates (λexc = 485 nm, λem = 528 nm) with a Synergy HTX Plate Reader (Biotek, Winooski, VT, USA). The fluorescence intensity (%) was expressed as relative fluorescence (F) measured in each sample normalized to the fluorescence of uncomplexed (naked) pDNA according to the following equation [28].

$$\mathrm{Fluorescence}\;\mathrm{intensity}\;\left(\%\right)=\frac{F\;\mathrm{sample}}{F\;\mathrm{naked}\;\mathrm{DNA}}\times 100$$

### 2.7. Transmission Electron Microscopy (TEM)

Niosome dispersions (5 μL) were placed on carbon coated grids and dyed with uranyl acetate (2% *w*/*v* in water). After being dried, the samples were observed using a high-resolution JEM-1010 TEM (JEOL USA Inc., Peabody, MA, USA).

### 2.8. hMSCs Isolation and Culture

Bone marrow aspirates were obtained from the proximal femur of patients undergoing hip arthroplasty (*n* = 5) provided by the Biobanco of A Coruña from SERGAS. The study was approved by the Comité de Ética de Investigación da Coruña (accession number: 2019/066). All patients provided informed consent before inclusion in the study. Human MSCs (hMSCs) were isolated and expanded in culture using standard protocols [33] and maintained in DMEM, 10% FBS, 100 U/mL penicillin G, 100 µL/mL streptomycin (growth medium) until use. All experiments were performed in hMSCs at passages 0–1.

### 2.9. hMSCs Characterization

hMSCs were characterized by Fluorescence Activated Cell Sorting (FACS). Briefly, cells were trypsinized, washed with FACS buffer and incubated at 4 °C for 45 minutes with antibodies: fluorescein isothiocyanate (FITC) isotype (1:50), phycoerythrin (PE) isotype (1:50), PE-conjugated anti-human CD34 (1:25), FITC-conjugated anti-human CD45 (1:25), PE-conjugated anti-human CD73 (1:25), FITC-conjugated anti-human CD90 (1:25) and FITC-conjugated anti-human CD105 (1:5). After incubation, cells were washed, resuspended in FACS buffer, and transferred to polypropylene tubes (NUNC, VWR International, Radnor, PA, USA). Acquisition was made with a BD FACS Calibur flow cytometer (BD Biosciences, Madrid, Spain), and data obtained was analysed using BD CellQuest Pro software (BD Biosciences, Madrid, Spain). For each assay, a minimum of 105 cell events were acquired and analysed [34].

### 2.10. Evaluation of Gene Transfer Efficiency Using P80 and P80PX Niosome Formulations

hMSCs were seeded in 48 well-plates at an initial density of 5 × 10^4^ cells/well and allowed to attach for 24 h at 37 °C before the experiments. Cells were exposed to p*lacZ* or pGFP nioplexes prepared as described in Section 2.3 (always 700 ng of plasmid). hMSCs cultured in Opti-MEM without nioplexes, and cells transfected with Lipofectamine™ (LPF; 1 μL/well) were used as negative and positive controls, respectively. Cells were incubated with nioplexes or LPF-based lipoplexes for 3 h at 37 °C and 5% CO_2_. After this time, the medium was removed and refreshed with growth medium, and cells were allowed to grow for 48 h at 37 °C, until being analysed. All conditions were assessed in triplicate in two independent experiments with cells isolated from two different patients.

The transfection efficiency of hMSCs achieved with p*lacZ* nioplexes was evaluated by using the β-glo reagent. Luminescence measurements were performed in white polystyrene 96 well-plates using a Synergy HTX Plate Reader and β-galactosidase activity was expressed as Relative Luminescence Units (RLU) [35]. Endogenous β-galactosidase activity recorded for the negative control was used as blank and subtracted from each condition and group of study.

β-galactosidase =RLU sample−RLU negative control

Transfection efficiency of pGFP nioplexes was evaluated by fluorescence microscopy. Briefly, a Hoechst 33342 dye solution (1 µg/mL) was added to each well and incubated for 10 min at room temperature. Then, cells were washed 2 times with PBS and observed under a fluorescence inverted microscope Olympus CKX53 (Olympus, Barcelona, Spain) with green fluorescence (GFP) and UV (Hoechst) filters sets. The percentage of transfected cells was determined by using ImageJ software to calculate the number of GFP-positive cells relative to the total number of cells from each condition evaluated.

$$\mathrm{pGFP}\;\mathrm{transfection}\;\mathrm{efficiency}\;\left(\%\right)=\frac{\mathrm{number}\;\mathrm{of}\;\mathrm{GFP}\;\mathrm{positive}\;\mathrm{cells}}{\mathrm{total}\;\mathrm{number}\;\mathrm{of}\;\mathrm{cells}}\times 100$$

### 2.11. Assessment of Cell Viability Using P80 and P80PX Niosome Formulations

Viability of hMSC monolayers upon contact with the different p*lacZ* nioplexes (same formulations and w/w ratios described in 2.10) was monitored at 48 h post-transfection using the tetrazolium salt (WST-1) method. Absorbance (A) at 450 nm was measured using a Synergy HTX Plate Reader and the percentage of cell viability (%) was calculated using the following equation [36]:

$$\mathrm{Viability}\;\left(\%\right)=\frac{A\;\mathrm{sample}}{A\;\mathrm{negative}\;\mathrm{control}}\times 100$$

Viability of hMSC monolayers transfected with the pGFP nioplexes was evaluated by comparing the total number of cells counted upon contact with the different formulations to the total cell number counted in the negative control (untransfected cells):

$$\mathrm{Viability}\;\left(\%\right)=\frac{\mathrm{number}\;\mathrm{of}\;\mathrm{cells}\;\mathrm{in}\;\mathrm{sample}}{\mathrm{number}\;\mathrm{of}\;\mathrm{cells}\;\mathrm{in}\;\mathrm{negative}\;\mathrm{control}}\times 100$$

### 2.12. Internalization Mechanism of P80 and P80PX Nioplexes

The endocytosis mechanisms of nioplexes were evaluated by β-galactosidase activity measurements after treatment with various endocytosis inhibitors at an optimized dose over time [37]. For that, hMSCs were plated in 96-well plates at 1.5 × 10^4^ cells/well and incubated for 24 h prior to the experiments. Each group of cells (triplicates) was pre-treated with different inhibitors: chlorpromazine as clathrin-mediated endocytosis inhibitor (30 µM, 1 h), genistein as caveolae-mediated endocytosis inhibitor (200 µM, 1 h), methyl-β-cyclodextrin as clathrin- and caveolae-dependent endocytosis inhibitor (2 mM, 10 min) and amiloride as macropinocytosis inhibitor (5 mM, 10 min). After pre-treatment, P80PX nioplexes were added and incubated for 4 h. Control conditions included cells transfected with P80PX nioplexes without inhibitor treatment. β-galactosidase activity and cell viability were measured following the same protocols described in Section 2.10 and Section 2.11, respectively. The uptake mechanism of nioplexes in hMSCs was further confirmed by confocal microscopy. For this, colocalization studies with Cy3-labeled nioplexes and AlexaFluor488-Cholera Toxin (10 μg/mL) or AlexaFluor488-Transferrin (50 μg/mL), which are markers of caveolae raft- and clathrin-mediated endocytosis respectively, were performed [38]. Briefly, hMSCs were seeded in 8-well µ-chamber slides (10^4^ cells/well) and co-incubated with Cy3-labeled nioplexes in the presence of AlexaFluor488-Cholera Toxin or AlexaFluor488-Transferrin for 2 h. Next, the medium containing nioplexes was removed, cells were washed twice with PBS and fixed with a 4% paraformaldehyde solution. Finally, nuclei were labeled with Hoechst 33342. Untransfected cells (negative control) were assessed in parallel. Cell fluorescence was visualized with an AR1 confocal microscope (Nikon, Tokio, Japan).

### 2.13. Chondrogenic Differentiation of hMSCs Using P80PX/psox9 Nioplexes

hMSCs (2 × 10^5^ cells) were pelleted to form aggregate cultures and incubated in defined chondrogenic medium in static conditions [33,39]. P80PX niosomes were complexed with the plasmid p*sox9* (P80PX/p*sox9*; 1 µg plasmid; cationic lipid mass ratio of 10/1) following the same procedure described in 2.10 and added to the cell aggregates before adding the chondrogenic medium. Positive control conditions included aggregates transfected with the same dose of plasmid complexed with the commercial reagent LPF (1 μL). Negative control conditions included untransfected pellets cultured in chondrogenic medium. All aggregates were cultured for 21 days at 37 °C.

#### 2.13.1. Histological and Immunohistochemical Analysis

hMSC aggregates were harvested, fixed, dehydrated and embedded in paraffin using standard protocols [33,39]. Paraffin-embedded sections (4 µm) were processed for staining with toluidine blue (matrix proteoglycans) and hematoxylin/eosin (H&E) to stain cell nucleus and cytoplasm [40]. Expression of SOX9, type-I, type-II, and type-X collagen were detected by immunohistochemistry using specific antibodies, biotinylated secondary antibodies, and the ABC method with diaminobenzidine as the chromogen [19,41]. To control for secondary immunoglobulins, samples were processed with omission of the primary antibody. All samples were examined under light microscopy Olympus CKX53.

#### 2.13.2. Histomorphometry

The cell densities (cell number/mm^2^) in H&E-stained sections and the intensities of toluidine blue staining and those of SOX9, type-I, type-II and type-X collagen immunostaining (all in pixels per standardized area) were measured at three random standardized sites (magnification 20×) for condition and replicate using the CellSens program and ImageJ.

#### 2.13.3. Total RNA Extraction and Real-Time RT-PCR Analyses

Total cellular RNA was extracted from aggregates using the RNeasy Protect Mini Kit with an on-column RNase-free DNase treatment. Reverse-transcription was carried out with 8 µL of eluate by using the 1st Strand cDNA Synthesis kit for RT-PCR (AMV) and 2.5 µL (125 ng/µL) of cDNA product was amplified with real-time PCR using the Brilliant SYBR Green qPCR Master Mix on a Lightcycler 480 QPCR operator (Roche, Madrid, Spain) under the following conditions: Denaturation (95 °C, 30 s), amplification by 40 cycles (denaturation at 95 °C, 5 s; annealing at 60 °C, 50 s; extension at 60 °C, 50 s) and denaturation (65–95 °C with an increment of 0.2 °C/s). The primers (Life Technologies ThermoFisher, Madrid, Spain) used were aggrecan (ACAN) (chondrogenic marker for proteoglycans) (forward 5-GAGATGGAGGGTGAGGTC-30; reverse 5-ACGCTGCCTCGGGCTTC-3′), SOX9 (early chondrogenic transcription factor) (forward 5′-ACACACAGCTCACTCGACCTTG-3′), type-II collagen (COL2A1) (chondrogenic and matrix marker) (forward 5′-GGACTTTTCTCCCCTCTCT-3′; reverse 5′-GACCCGAAGGTCTTACAGGA-3′), type-I collagen (COL1A1) (osteogenic marker) (forward 5′-ACGTCCTGGTGAAGTTGGTC-3′; reverse 5′-ACCAGGGAAGCCTCTCTCTC-3′), type-X collagen (COL10A1) (marker of hypertrophy) (forward 5′-CCCTCTTGTTAGTGCCAACC-3′; reverse 5′-AGATTCCAGTCCTTGGGTCA-3′), and glyceraldehyde-3-phosphate dehydrogenase (GAPDH) (housekeeping gene and internal control) (forward 5′-GAAGGTGAAGGTCGGAGTC-3′; reverse 5′-GAAGATGGTGATGGGATTTC-3′) (all at 300 nM final concentration). The threshold cycle (Ct) value for each gene of interest was measured for each amplified sample by using the BioRad CFX Maestro software and normalized to GAPDH expression. Fold inductions (relative to untransfected control aggregates) were calculated by using the 2^−ΔΔCt^ method, as previously depicted [33,39].

#### 2.13.4. Biochemical Analysis

hMSC aggregates were digested with papain (125 µg/mL, pH 6.5) at 60 °C for 1 h as previously described [39]. The proteoglycan contents were estimated by binding to dimethylmethylene blue dye (DMMB) using chondroitin sulfate as standard. Data were normalized to the total protein contents using a bicinchoninic acid (BCA) assay or to the DNA contents by the Hoechst 33342 assay [42]. All measurements were performed by triplicate on a Synergy HTX Plate Reader.

### 2.14. Statistical Analysis

Data were expressed as mean ± standard deviation (SD) of three technical replicates from each patient. A total of five patients (n = 5) were involved for the different studies as follows: evaluation of gene transfer efficiency (n = 2), internalization mechanisms (n = 1) and chondrogenic differentiation (n = 2). Statistical analysis was performed using IBM SPSS Statistics version 23 by parametric tests (one-way ANOVA; Student’s *t*-test) and non-parametric ones (Kruskal-Wallis, Multiple Range, Mann–Whitney U), when appropriate in each case. A *p* ≤ 0.05 was considered statistically significant.

## 3. Results

### 3.1. Physicochemical Characterization of Niosomes and Nioplexes

Niosomes were prepared by a reverse evaporation technique using a single (P80) or a mixture of surfactants (P80PX) as main components, together with squalene and DOTMA as helper and cationic lipid, respectively.

**Figure 1:**
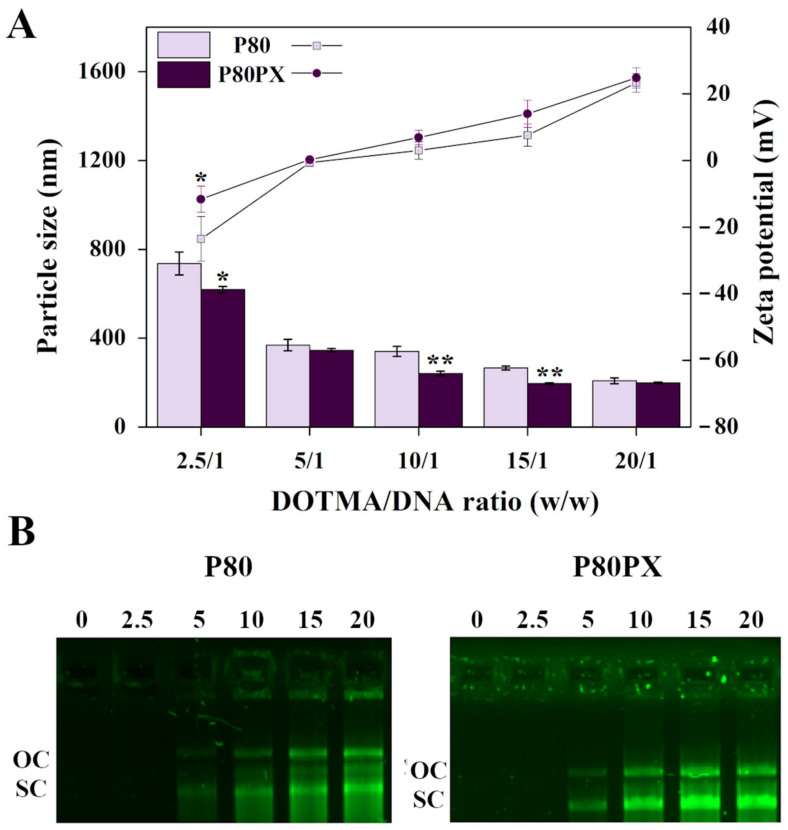
(**A**) Particle size and zeta potential. (**B**) DNase protection ability and SDS-induced release visualized by agarose electrophoresis of polysorbate 80 (P80, mauve squares) and polysorbate 80 combined with poloxamer 407 (P80PX, dark purple circles); nioplexes formed at DOTMA/DNA mass ratios of 2.5/1, 5/1, 10/1, 15/1 and 20/1. 0: naked pl*acZ*; OC: open circular; SC: supercoiled * depicts *p* < 0.05 and ** *p* < 0.01 when comparing both nioplexe formulations at the same DOTMA/DNA ratio.

Niosomes were first characterized in terms of size, zeta potential (Figure 1A) and PDI (Table S1 Supplementary Materials). The size of niosomes formulated with P80PX (223.2 ± 8.9 nm) was significantly lower (*p* = 0.025) than that recorded with niosomes formulated with P80 solely (310.8 ± 40.3 nm). Representative TEM images of P80 and P80PX niosomes are shown in Figure S1. Surface charges were 24.4 ± 3.0 mV and 25.6 ± 2.2 mV for P80 and P80PX, respectively, without differences between both formulations (*p* = 0.37). Nioplexes were formed upon complexation of p*lacZ* with P80 or P80PX niosomes at DOTMA/DNA mass ratios of 2.5/1, 5/1, 10/1, 15/1 and 20/1. Irrespective of the DOTMA/DNA *w*/*w* ratio considered, and similarly to that observed with niosome formulations, the size of nioplexes formulated with P80PX was generally lower than those containing P80 solely, although not statistically significant differences were reached (*p* ≥ 0.300) (Figure 1A).

Focusing on DOTMA/DNA mass ratios, nioplexes formed at the lowest ratio (2.5/1) and showed the biggest sizes, being around 736.4 ± 51.7 nm for P80 and 618.9 ± 14.5 nm for P80PX ones (always *p* ≤ 0.010 compared with nioplexes formed at the rest of mass ratios). Both nioplexe formulations showed a size decrease with the raise of DOTMA/DNA ratio, from 369.2 ± 25.4 nm at 5/1 mass ratio to 208.5 ± 13.3 nm at 20/1 mass ratio in P80 nioplexes, and from 345.7 ± 8.0 nm to 199.0 ± 2.9 nm with the same ratios of P80PX complexes (*p* ≤ 0.007).

A similar trend was observed for zeta potential values of nioplexes, increasing their electropositivity from −23.5 ± 6.7 mV at 2.5/1 DOTMA/DNA ratio up to 23.3 ± 2.8 mV at 20/1 DOTMA/DNA ratio for P80 nioplexes (*p* = 0.004), and from −11.6 ± 3.9 mV up to 24.8 ± 3.0 mV at similar ratios for the P80PX ones (*p* = 0.006).

Polydispersity index (PDI) values were around 0.3 for P80 and P80PX nioplexes without differences between ratios from both formulations (*p* ≥ 0.229).

Agarose gel electrophoresis assays in the presence of DNase I are summarized in Figure 1B. In contrast to that observed with free plasmid (0), an enhanced protection capacity was noticed by increasing the DOTMA/DNA ratio from 2.5/1 to 20/1 in both P80 (left panel) and P80PX (right panel) nioplexes. After the addition of SDS, both nioplexe formulations showed SC (supercoiled) and OC (open circular) bands corresponding to the released DNA.

### 3.2. Complexation of DNA with P80 or P80PX Niosome Formulations

**Figure 2:**
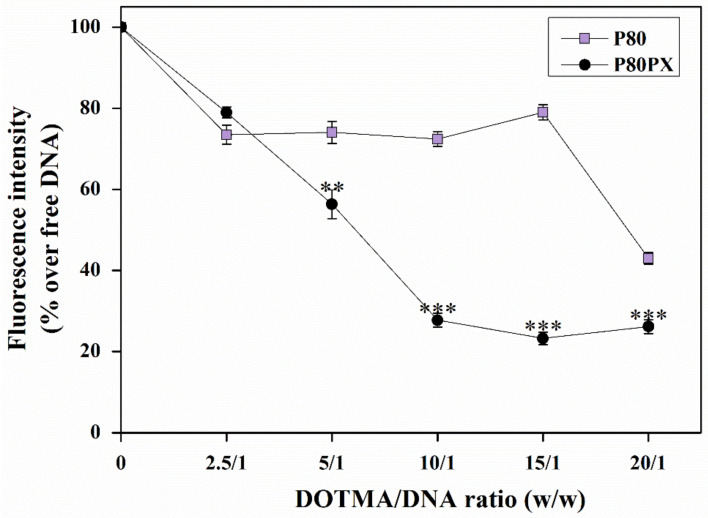
DNA complexation efficiency of P80 (mauve squares) and P80PX (dark purple circles) nioplexes formed at DOTMA/DNA mass ratios of 2.5/1, 5/1, 10/1, 15/1 and 20/1. ** depicts *p* < 0.01 and *** *p* < 0.001 when comparing both nioplexe formulations at the same DOTMA/DNA ratio.

The ability of the P80 and P80PX formulations to efficiently condense p*lacZ* was then evaluated, by applying the SYBR green dye exclusion assay (Figure 2). Both nioplexe formulations led to a decrease in fluorescence values with the raise of DOTMA/DNA mass ratios reaching the highest efficiency with those nioplexes formulated with both non-ionic surfactants (*p* ≤ 0.04).

Complexation ability of P80 nioplexes showed a lineal tendency from 2.5/1 to 15/1 DOTMA/DNA ratio without differences between DOTMA/DNA ratios (~30% p*lacZ* complexation efficiency; *p* ≤ 0.151), exhibiting a sharp decrease at a 20/1 ratio (~57% p*lacZ* complexation efficiency; *p* = 0.010). Conversely, P80PX nioplexes showed a gradual decrease on fluorescence values from 2.5/1, reaching the maximal complexation ability at a 15/1 DOTMA/DNA ratio (~76% p*lacZ* complexation; *p* < 0.001) without further increase on complexation ability afterwards (*p* = 0.028) [32].

### 3.3. In Vitro Transfection of hMSCs

In vitro transfection assays with P80 or P80PX nioplexes (cationic lipid/DNA 5/1, 10/1, 15/1 and 20/1 w/w ratios) were performed in hMSC primary cultures isolated from bone marrow samples. Isolated cells showed the specific profiles of expression characteristic from hMSCs (Table S2 and Figure S2 Supplementary Materials). In all cases, cells showed mean positivity percentages >69% for the three MSC-positive surface markers (CD90, CD73, and CD105). The expression of CD90 and CD73 was >93%, although the positivity for CD105 was slightly lower (~70%). Conversely, expression of hematopoietic surface markers (CD34 and CD45) showed values <1% (Table S2 and Figure S2 Supplementary Materials).

**Figure 3:**
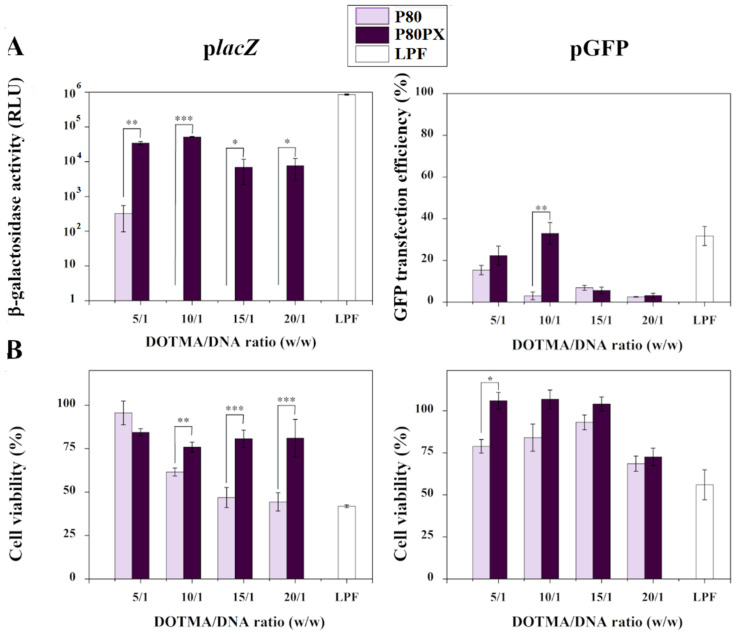
(**A**) β-galactosidase activity (left), pGFP transfection efficiency (right) and (**B**) Cell viability of P80 (mauve) and P80PX (dark purple) nioplexes formed at DOTMA/DNA mass ratios of 2.5/1, 5/1, 10/1, 15/1 and 20/1. The commercial reagent Lipofectamine (LPF) was used as positive transfection control. * depicts *p* < 0.05, ** *p* < 0.01 and *** *p* < 0.001, when compared with denoted groups.

Transfection efficiency and cell viability of hMSCs upon contact with P80 or P80PX nioplexes are summarized in Figure 3. Gene transfer efficiency of hMSC monolayers treated with p*lacZ* nioplexes always showed a superior efficiency when using P80PX formulation compared with those comprising P80 solely, at all DOTMA/DNA ratios studied (*p* ≤ 0.034) (Figure 3A). Of note, P80 nioplexes only resulted in measurable values of β-galactosidase activity when using nioplexes at the lowest DOTMA/DNA ratio (5/1) (321 ± 26 RLU), being significantly lower to those achieved with P80PX nioplexes (always *p* ≤ 0.006). In contrast, P80PX nioplexes led to an effective transfection at all DOTMA/DNA ratios tested, especially at 10/1 DOTMA/DNA ratio (51,321 ± 2206 RLU), being still lower to that achieved with the commercial reagent Lipofectamine (854,598 ± 37,401 RLU) (*p* = 0.029).

A similar trend was observed when using pGFP nioplexes, although only statistically significant differences were reached when using nioplexes at 10/1 DOTMA/DNA mass ratio (32.9 ± 5.2 % GFP positive cells) (*p* < 0.01). Of note, transfection with P80PX nioplexes led to similar values of gene transfer to those achieved with the commercial reagent Lipofectamine (31.7 ± 4.6 % GFP positive cells) (*p* ≥ 0.372) (Figure 3A and Figure S3 Supplementary Materials).

Of note, percentages of cell survival were markedly higher upon transfection with P80PX than those obtained with P80 nioplexes at most of the DOTMA/DNA ratios tested, showing the first one percentage ranging from 100% to 73% at the highest DOTMA/DNA ratio tested (Figure 3B). Moreover, these percentages were remarkably superior to those achieved with LPF especially at the 10/1 DOTMA/DNA ratio (up to 1.8-fold difference *p* < 0.001).

### 3.4. Cell Internalization of Nioplexes

In order to evaluate the endocytosis mechanisms of niosomes in hMSCs, we quantified the transgene expression (*lacZ*) of P80PX nioplexes at a 10/1 DOTMA/DNA ratio after treatment with different inhibitors by luminescence measurements (Figure S4A Supplementary Materials). A significant reduction on β-galactosidase activity was evidenced in those cells pre-treated with chlorpromazine (21,029 ± 4369 RLU) and methyl-β-cyclodextrin (33,825 ± 12,085 RLU) when compared with untreated control cells (99,072 ± 5646 RLU) (*p* < 0.02). These results pointed out that nioplexes predominantly enter by clathrin-mediated endocytosis (Figure S4A Supplementary Materials).

These findings were further confirmed by confocal microscopy of Cy3-labeled p*lacZ* P80PX nioplexes in hMSC cultures (Figure S4B Supplementary Materials). The co-localization of Cy3-labeled p*lacZ* nioplexes with transferrin was higher than that observed with cholera toxin (Figure S4B Supplementary Materials), reiterating a preferential internalization by clathrin-mediated endocytosis.

### 3.5. Chondrogenic Differentiation of hMSCs upon Transfection with P80PX/psox9 Nioplexes

hMSC aggregates were transfected with P80PX/p*sox9* nioplexes and cultured in chondrogenic medium for 21 days. Control conditions included untransfected aggregates cultured in chondrogenic medium (negative control: control) or cultured in the same medium but transfected with p*sox9* using Lipofectamine reagent (positive control: LPF).

**Figure 4:**
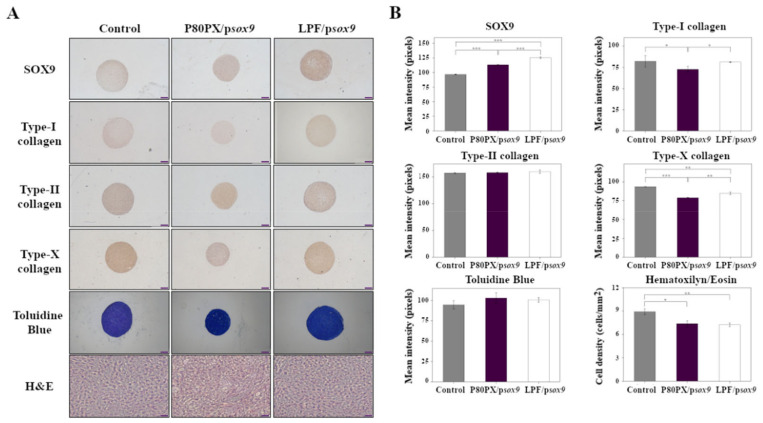
Immunohistochemical and histological analyses of hMSC aggregates cultured in chondrogenic medium (control; negative control) and transfected with p*sox9* via P80PX (P80PX/p*sox9*) or LPF (LPF/p*sox9*). Samples were kept in culture for 21 days and processed for (**A**) Immunodetection of SOX9, type-I, type-II and type-X collagen, and toluidine blue (all representative images; magnification 4X; scale bar 50 µm) or Hematoxylin/Eosin (H&E) stainings (all representative images; magnification 10x; scale bar 100 µm). (**B**) Histomorphometrical analyses (control: grey; P80PX/p*sox9*: dark purple; LPF/p*sox9*: white) realized as described in Materials and Methods (Section 2.13.2; Figure S5). * depicts *p* < 0.05, ** *p* < 0.01 and *** *p* < 0.001, when compared with denoted groups.

**Figure 5:**
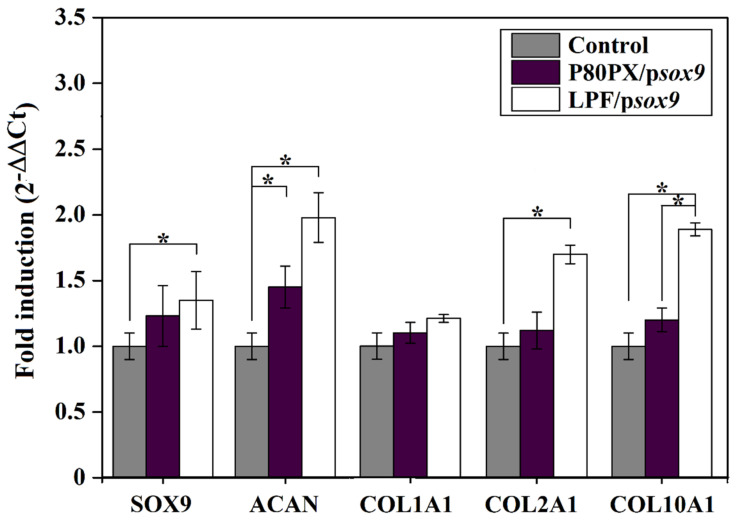
Real time RT-PCR analysis of hMSC aggregates cultured in chondrogenic medium (control; negative control: grey) and transfected with p*sox9* via P80PX (P80PX/p*sox9*: dark purple) or LPF (LPF/p*sox9*: white) complexes after 3 weeks in vitro. The genes analysed included aggrecan (ACAN), the transcription factor SOX9, type-II collagen (COL2A1), type-X collagen (COL10A1) and type I collagen (COL1A1) with GAPDH serving as a housekeeping gene and internal control (primers are listed in Section 2.13.3). Ct values were obtained for each target and for GAPDH as a control for normalization, and fold inductions (relative to control aggregates) were measured by using the 2^−ΔΔCt^ method. * depicts *p* < 0.05 when compared P80PX/p*sox9* and P80PX/LPF groups with control group.

Transgene (SOX9) expression was noted in all hMSC aggregates, as a probable result of the effective differentiation of the cells under continuous chondrogenic induction and particularly when p*sox9* was provided to the cells (Figure 4A). Still, expression of the chondrogenic factor was always higher following *sox9* gene transfer via LPF lipoplexes (125.3 ± 1.2 pixels) or P80PX nioplexes (113.2 ± 0.8 pixels) when compared with untransfected aggregates (*p* ≤ 0.003) (Figure 4B). These findings were corroborated by an analysis of *sox9* profiles by real-time RT-PCR, although statistical significance was only reached using LPF/p*sox9* complexes (1.6-fold increase, *p* = 0.04) (Figure 5).

Chondrogenic differentiation was noted in all the samples as noted by the intense toluidine blue staining (matrix proteoglycans), and type-II collagen deposition (Figure 4A), especially in those aggregates transfected with P80PX/p*sox9* (102.9 ± 6.5 and 158.5 ± 0.2 pixels, respectively) or LPF/p*sox9* (100.7 ± 2.7 and 160.2 ± 3.3 pixels, respectively) complexes although no statistical differences between groups were observed (Figure 4B). Yet, real-time RT-PCR analyses showed an up-regulation of ACAN in cell aggregates transfected with LPF/p*sox9* (2.0-fold increase) or P80PX/p*sox9* (1.4-fold increase) complexes when compared with control untransfected cells (*p* < 0.05). Up-regulation of COLII expression was also evidenced upon SOX9 overexpression, although only LPF/p*sox9* (1.7-fold increase) complexes showed statistically significant differences compared with the control (*p* = 0.04).

Of note, genetic modification of aggregates via P80PX/ps*ox9* nioplexes led to a significant reduction in COLI immunoreactivity (72.6 ± 3.4 pixels) when compared with those pellets transfected with LPF/p*sox9* (81.1 ± 0.4 pixels) or with control aggregates (81.9 ± 6.5 pixels) *(p ≤* 0.034) (Figure 4A, B). However, no differences between groups in this marker were observed at the mRNA level (*p* ≤ 0.43) (Figure 5).

A significant reduction of type-X immunoreactivity was also evidenced in cell aggregates transfected with P80PX/p*sox9* (78.9 ± 0.5 pixels) nioplexes when compared with those pellets transfected with LPF/p*sox9* (84.7 ± 1.8 pixels) or with control untransfected cells (93.5 ± 0.3 pixels) (*p* ≤ 001) (Figure 4A,B). Further, these differences were confirmed by real-time RT-PCR analysis showing those aggregates transfected with P80PX/p*sox9* with significantly lower levels of this marker (1.2-fold induction) than LPF/p*sox9* transfected ones (1.9-fold induction) (*p* = 0.03) (Figure 5).

Lastly, an analysis of cellularity in cell aggregates by H&E staining evidenced lower cell densities in those pellets transfected with P80PX/p*sox9* (7.4 ± 0.4 pixels/mm^2^) or LPF/p*sox9* (7.2 ± 0.2 pixels/mm^2^) complexes compared with untransfected control ones (8.9 ± 0.4 pixels/mm^2^) (*p* ≤ 0.04).

**Figure 6:**
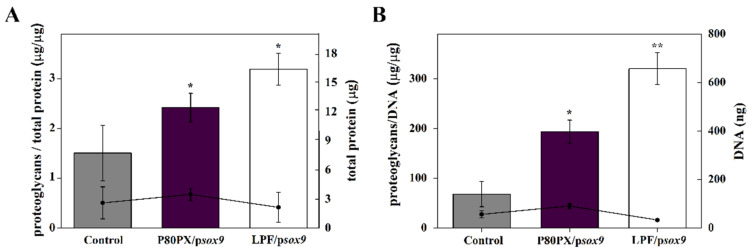
Biochemical analyses of hMSC aggregates cultured in chondrogenic medium (control; negative control: grey) and transfected with p*sox9* via P80PX (P80PX/p*sox9*: dark purple) or LPF (LPF/p*sox9*: white) complexes after 3 weeks in vitro. (**A**) Proteoglycan contents standardized to the protein contents and (**B**) Proteoglycan contents standardized to the DNA contents. * depicts *p* < 0.05 and ** *p* < 0.01 when compared P80PX/p*sox9* and P80PX/LPF groups with control.

These data were further corroborated by an analysis of the proteoglycan contents in the aggregate cultures standardized to the total protein (Figure 6A) or DNA contents (Figure 6B). SOX9 overexpression in hMSC aggregates led to an increase on proteoglycan contents compared with those pellets cultured in chondrogenic medium (control), up to 1.6-fold difference when using P80PX/p*sox9* nioplexes and up to 2.1-fold difference via LPF/p*sox9* lipoplexes (*p* ≤ 0.03) (Figure 6A). A similar trend was observed by normalizing the number of proteoglycans to the DNA contents (Figure 6B), leading those pellets transfected with P80PX/p*sox9* nioplexes and LPF/p*sox9* lipoplexes to 2.9- and 4.5-fold differences compared with aggregates cultured in chondrogenic medium, respectively (*p* ≤ 0.022). In good agreement with viability studies (Figure 3B), transfection via LPF/p*sox9* lipoplexes resulted in a reduction of both protein (2.1 ± 1.5 µg) and DNA (32.5 ± 2.9 ng) contents when compared with untransfected (2.6 ± 1.6 µg and 55.8 ±14.7 ng) or transfected aggregates with P80PX/p*sox9* nioplexes (3.5 ± 0.6 µg and 90.4 ± 10.1 ng), although only statistically significant differences were reached in DNA contents (*p* ≤ 0.02).

## 4. Discussion

Direct local genetic modification of hMSCs constitutes a powerful strategy to maximize the potency of this valuable progenitor cell population towards proper chondrocyte differentiation [33]. Among current gene transfer vectors to promote MSCs chondrogenesis, viral vectors represent gold standard vehicles due to their superior gene transfer efficiency when compared with their non-viral counterparts. Nonetheless, the use of these vectors is still precluded by important limitations including high immunogenicity and/or toxicity and the possibility of insertional mutagenesis. Herein, the design of new adapted non-viral vectors for efficient gene transfer of MSCs is at the forefront of gene therapy.

Niosomes have recently emerged as attractive gene transfer tools with a marked efficiency to transfect retina [43,44] or primary neuronal cells [45], exhibiting important advantages over classical liposomes [28]. However, only a few studies up to date have described the possibility of using niosomes as delivery systems to genetically modify murine MSCs [2,46] or human immortalized MSCs [28] with reporter [28,46] or osteogenic [2] genes in various regenerative medicine approaches. Further, none of these studies has tackled the possibility of employing niosomes as gene carriers to promote hMSCs chondrogenesis.

Thereupon, in the present study, we examined for the first time the ability of niosomes as gene transfer vehicles to promote chondrogenic differentiation of hMSCs.

The data from the study first revealed an influence of the non-ionic surfactant composition on physicochemical and complexation ability of resulting niosomes. Niosomes and resulting nioplexes formulated with both non-ionic surfactants (P80PX) showed lower sizes and higher electropositivity. These results are in agreement with previous studies showing a reduction of size on niosomes formulated with the cationic lipid 2,3-di(tetradecyloxy)propan-1-amine and a mixture of P80 and PX non-ionic surfactants with or without the helper lipid chloroquine [45,46]. Moreover, in good concordance with the lower sizes measured with P80PX nioplexes, the complexation ability of P80PX niosome formulations was significantly higher than that achieved with P80 ones. In addition, agarose gel electrophoresis assays revealed that both niosome formulations were able to condense, release and protect DNA from enzymatic digestion against DNase, as confirmed by the presence of SC bands [28].

In order to test the performance of developed formulations for gene delivery purposes, transfection assays were carried out in primary cultures of hMSCs isolated from bone marrow of different patients. Isolated cells showed proper characteristics of MSCs according to criteria stated by the International Society of Cellular Therapy [47]. Of note, reporter gene transfer efficiencies (*lacZ*, GFP) of hMSCs via P80PX nioplexes were significantly higher to those achieved with nioplexes comprising only P80 surfactant, especially at a cationic DOTMA/DNA ratio of 10/1. A similar trend was reported by Beitia et al. noting an increment of luciferase expression in mouse bone marrow derived MSCs by combining both surfactants in niosome formulations [46]. Increase of gene expression levels in the presence of PX may be attributed to the higher hydrophilic-lipophilic balance (HLB) of this non-ionic surfactant (HLB = 18–23) compared with P80 (HLB = 15), resulting in a higher stabilization of complexes and in an increased transfection [48].

Likewise, percentages of cell survival were significantly higher upon transfection with P80PX nioplexes when compared with P80 ones, being more evident at the highest DOTMA/DNA ratios tested. This fact may be due to the protective effect of poloxamer on the cell membrane leading to a preservation of cell viability [49,50] when compared with P80 nioplexes exhibiting a higher cytotoxicity upon contact with MSCs [2]. Of note, P80PX nioplexes led to significantly higher levels of cell survival to those achieved with the gold standard Lipofectamine, which showed a clear reduction in MSCs viability and was in good agreement with previous observations [28,51].

As niosomes’ composition has a strong impact in its ability for transfection, depending on the cell type and the specific cell uptake pathway [31], internalization studies were performed with P80PX nioplexes at a DOTMA/DNA ratio of 10/1. Quantitative and qualitative assays showed a preferential internalization of P80PX nioplexes by clathrin-mediated endocytosis in hMSCs being the main pathway for cationic-lipid based systems [52]. It is well known that genes internalized through clathrin-mediated endocytosis need to escape from endosomes to avoid lysosomal digestion and get an efficient transgene expression [53]. As a matter of fact, previous observations showed an enhanced pDNA endosomal escape by using non-viral based formulations containing the helper lipid squalene [38] or the non-ionic surfactant poloxamer 407 [54]. Therefore, tuning niosomes composition is fundamental to enhance the release of nucleic acids from endosomes to cytoplasm, resulting in high transfection efficiencies.

In order to test the performance of P80PX niosomes to promote an effective chondrogenesis of hMSCs, systems were complexed with plasmid encoding for the potent chondrogenic transcription factor *sox9*. An effective SOX9 gene transfer was noted upon transfection of hMSCs with P80PX or LPF complexes, exhibiting higher levels of transgene expression than those cells cultured in chondrogenic medium (untransfected cells; control). Nonetheless, only the LPF group achieved statistically significant differences in both immunohistochemical and RT-PCR analyses. Chondrogenic differentiation of hMSCs in micromass cultures treated with TGF-β3 was evidenced in the three groups of study, as noticed by the production of cartilage specific, sulfated glycosaminoglycans and type-II collagen [55]. However, as SOX9 plays a pivotal role during early stages of chondrogenesis [17], formulation of nioplexes with higher dose per cell of p*sox9* [21] may show a higher expression of these key cartilage markers.

Remarkably, and in good agreement with previous works using viral vectors [19,56], overexpression of SOX9 via P80PX nioplexes reduced the expression and activities of markers of hypertrophy and terminal/or osteogenic differentiation (type-I and type-X collagen) [56] of MSCs at the protein level, as depicted in immunohistochemical analysis. Moreover, and also in consonance with previous observations [19,56], overexpression of SOX9 did not increase the cell densities (H&E staining) of hMSC aggregates.

Lastly, biochemical analyses revealed an increase in proteoglycans biosynthesis in those cells transfected with P80PX/p*sox9* or LPF/p*sox9* complexes, as compared with control untransfected cells. Of note, and in consonance with reporter gene transfection studies, p*sox9* transfer using Lipofectamine led to a decrease in both DNA and protein contents, compared with those aggregates transfected with P80PX/p*sox9* nioplexes.

## 5. Conclusions

Niosomes formulated with DOTMA as cationic lipid, squalene as helper lipid and polysorbate 80 solely (P80) or combined with poloxamer 407 (P80PX) as non-ionic surfactants were investigated for the first time as new systems to genetically modify MSCs. P80PX niosome formulations led to high transfection efficiency of reporter gene plasmids (*lacZ*, GFP) with a marked lower cytotoxicity when compared with P80 solely- or Lipofectamine-based complexes. Overexpression of the transcription factor SOX9 via these niosomal formulations led to an effective chondrogenesis of MSCs with a reduced hypertrophy. Further studies involving nioplexes with a higher dose of *sox9* may reveal a superior chondrogenic efficiency.

Overall, these findings show the potential benefits of P80PX niosomes as promising tools for gene transfer to MSCs in cartilage reparative approaches.

## Data Availability

Data available on request.

## Supplementary Materials

The following Supplementary Materials can be downloaded at: https://www.mdpi.com/article/10.3390/pharmaceutics14112327/s1, Table S1. Physical characterization of P80 and P80PX niosomes formulations. Mean values ± standard deviation (n = 3). Table S2. Flow cytometry analysis of CD45, CD34, CD90, CD73 and CD105 markers. Mean values ± standard deviation (n = 2), and where 105 events were acquired for each sample. Figure S1. Representative TEM images of A. P80 and B. P80PX niosomes formulations. Scale bar 0.5 µm. Figure S2. Representative images for the analysis of hMSCs surface markers. Flow cytometry analysis of CD45, CD34. CD90, CD73 and CD105 compared with their corresponding isotype FITC or PE as negative control (M1). Figure S3. Fluorescence microscopy representative images (magnification 4×; scale bar 50 µm) showing GFP expression (green) after transfection of hMSCs with P80 and P80PX nioplexes formed at DOTMA/DNA of 5/1, 10/1, 15/1 and 20/1. Cells cultured in Opti-MEM and cells transfected with Lipofectamine (LPF) were used as negative and positive control, respectively. Cell nuclei were counterstained with Hoechst 33342 (blue). Figure S4. (A) Cell viability (left) and β-galactosidase activity (right) of MSCs pre-treated or not (C: control) with endocytosis inhibitors: CP; chlorpromazine; GEN; genistein; AM; amiloride and MeCD; methyl-β-cyclodextrin) and subsequent transfection with P80PX nioplexes (10/1). (B) Confocal microscopy representative images (magnification 10x scale bar 100 µm) showing the intracellular distribution of P80PX nioplexes (10/1) in MSCs. Blue coloring shows cell nuclei stained with Hoechst 33342, red color shows Cy3-labeled-p*lacZ* nioplexes and green coloring shows cells stained with AlexaFluor488-Transferrin or AlexaFluor488-Cholera toxin. Figure S5. Representative images (magnification 20×; scale bar 50 µm) used in the histomorphometrical analysis of hMSCs aggregates cultured in chondrogenic medium (control; negative control) and transfected with psox9 via P80PX (P80PX/psox9) or LPF (LPF/psox9). Samples were kept in culture for 21 days and processed for immunodetection of SOX9, type-I, type-II and type-X collagen and toluidine blue.
