# Supplementary material for: Chondrogenic Differentiation of Human Mesenchymal Stem Cells via SOX9 Delivery in Cationic Niosomes
Source: Pharmaceutics. 2022 Oct 28;14(11):2327. doi: 10.3390/pharmaceutics14112327 (PMC9693355; doi:10.3390/pharmaceutics14112327)
Supplement: Supplementary file 1 [file pharmaceutics-14-02327-s001.zip › pharmaceutics-1966135-supplementary.pdf]

pharmaceutics

Supporting Information

Chondrogenic differentiation of human mesenchymal stem cells *via* SOX9 delivery in cationic niosomes

Natalia Carballo-Pedrares [1], Clara Sanjurjo-Rodriguez [2,3], Jose Señarís [3], Silvia Díaz-Prado [2,3] and Ana Rey Rico [1,*]

[1] Gene & Cell Therapy Unit (G-CEL), Centro de Investigacións Científicas Avanzadas (CICA), Universidade da Coruña, A Coruña, Spain
[2] Cell Therapy and Regenerative Medicine Unit, Centro de Investigacións Científicas Avanzadas (CICA), Universidade da Coruña, A Coruña, Spain
[3] Institute of Biomedical Research of A Coruña (INIBIC), University Hospital Complex A Coruña (CHUAC), Galician Health Service (SERGAS).
* Correspondence: Gene & Cell Therapy Unit (G-CEL). Centro de Investigacións Científicas Avanzadas (Universidade da Coruña), As Carballeiras, s/n. Campus de Elviña 15071 A Coruña, España; ana.rey.rico@udc.es; Tel.: +34 881 01 5543.

	Particle size (nm)	Zeta potential (mV)	PDI
**P80**	310.8 ± 40.3	24.4 ± 3.0	0.29 ± 0.05
**P80PX**	223.2 ± 8.9	25.6 ± 2.2	0.28 ± 0.03

Table S1. Physical characterization of P80 and P80PX niosomes formulations. Mean values ± standard deviation (n=3).

	Positivity surface markers (%)
**FICT-CD45**	0.90 ± 1.16
**PE-CD34**	0.86 ± 0.66
**FICT-CD90**	93.52 ± 6.48
**PE-CD73**	97.04 ± 0.49
**FICT- CD105**	69.80 ± 6.55

Table S2. Flow cytometry analysis of CD45, CD34. CD90, CD73, and CD105 markers. Mean values ± standard deviation (n=2), 10^5 events were acquired for each sample.

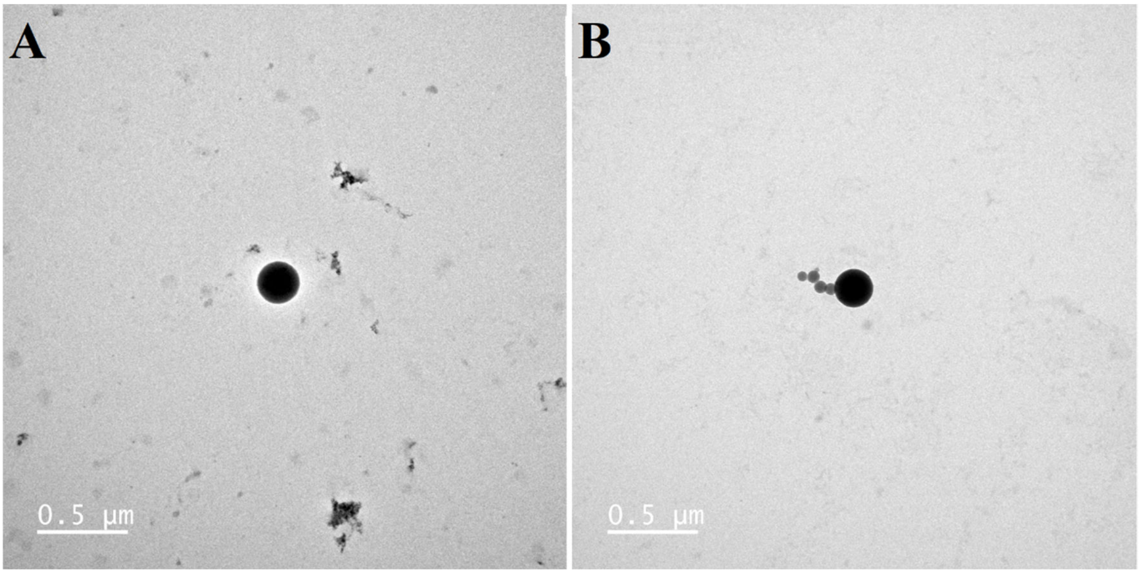

Figure S1. Representative TEM images of **A.** P80 and **B.** P80PX niosomes formulations. Scale bar 0.5 µm.

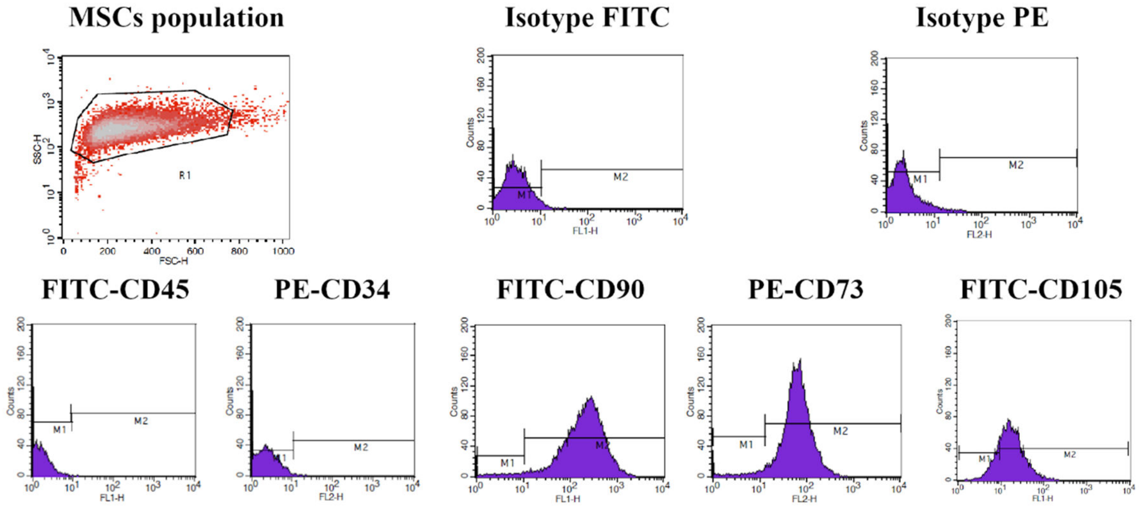

Figure S2. Representative images for the analysis of hMSCs surface markers. Flow cytometry analysis of CD45, CD34. CD90, CD73, and CD105 compared with their corresponding isotype FITC or PE as negative control (M1).

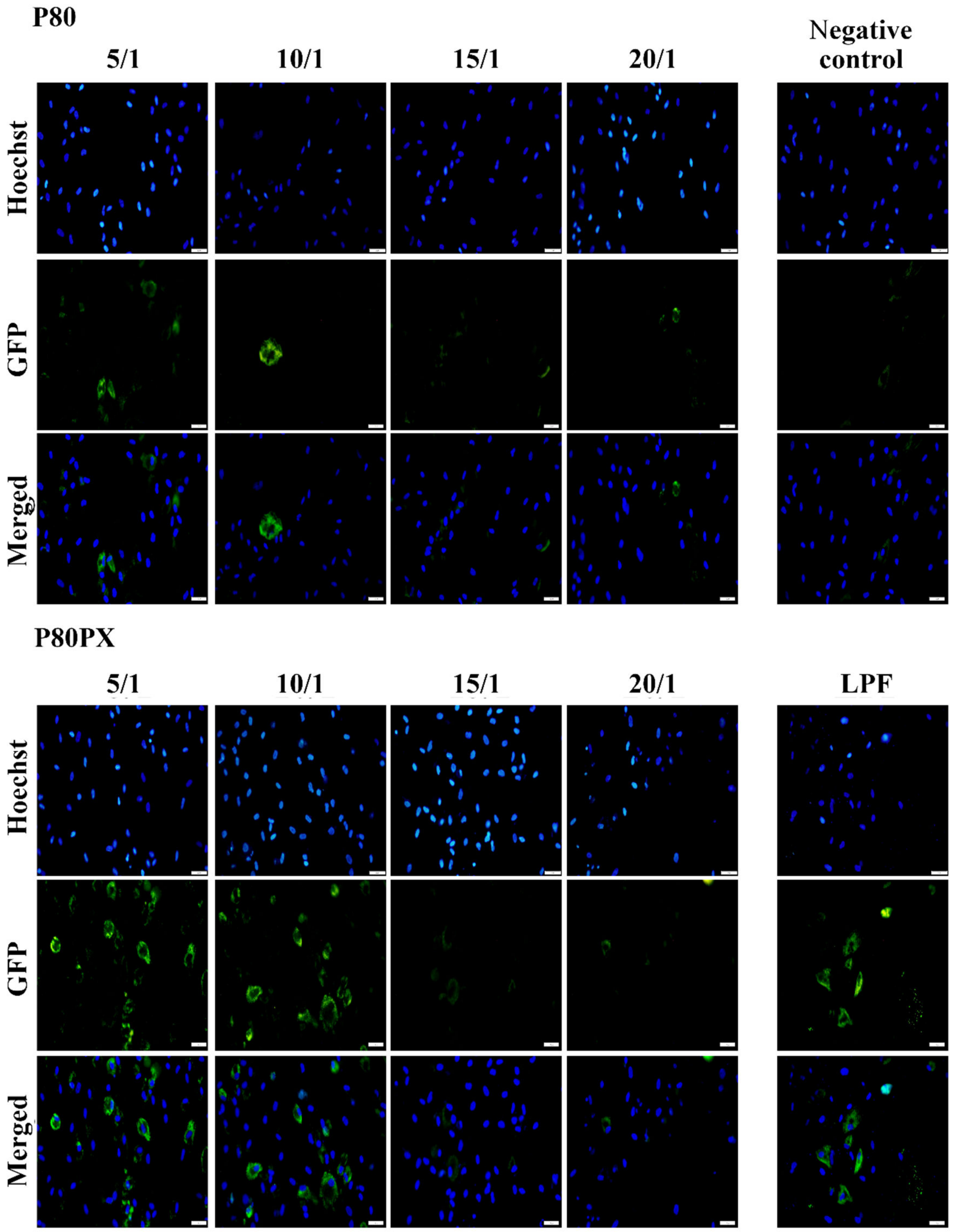

Figure S3. Fluorescence microscopy representative images (magnification 4x; scale bar 50 µm) showing GFP expression (green) after transfection of hMSCs with P80 and P80PX nioplexes formed at DOTMA/DNA of 5/1, 10/1, 15/1 and 20/1. Cells cultured in Opti-MEM and cells transfected with Lipofectamine (LPF) were used as negative and positive control, respectively. Cell nuclei were counterstained with Hoechst 33342 (blue).

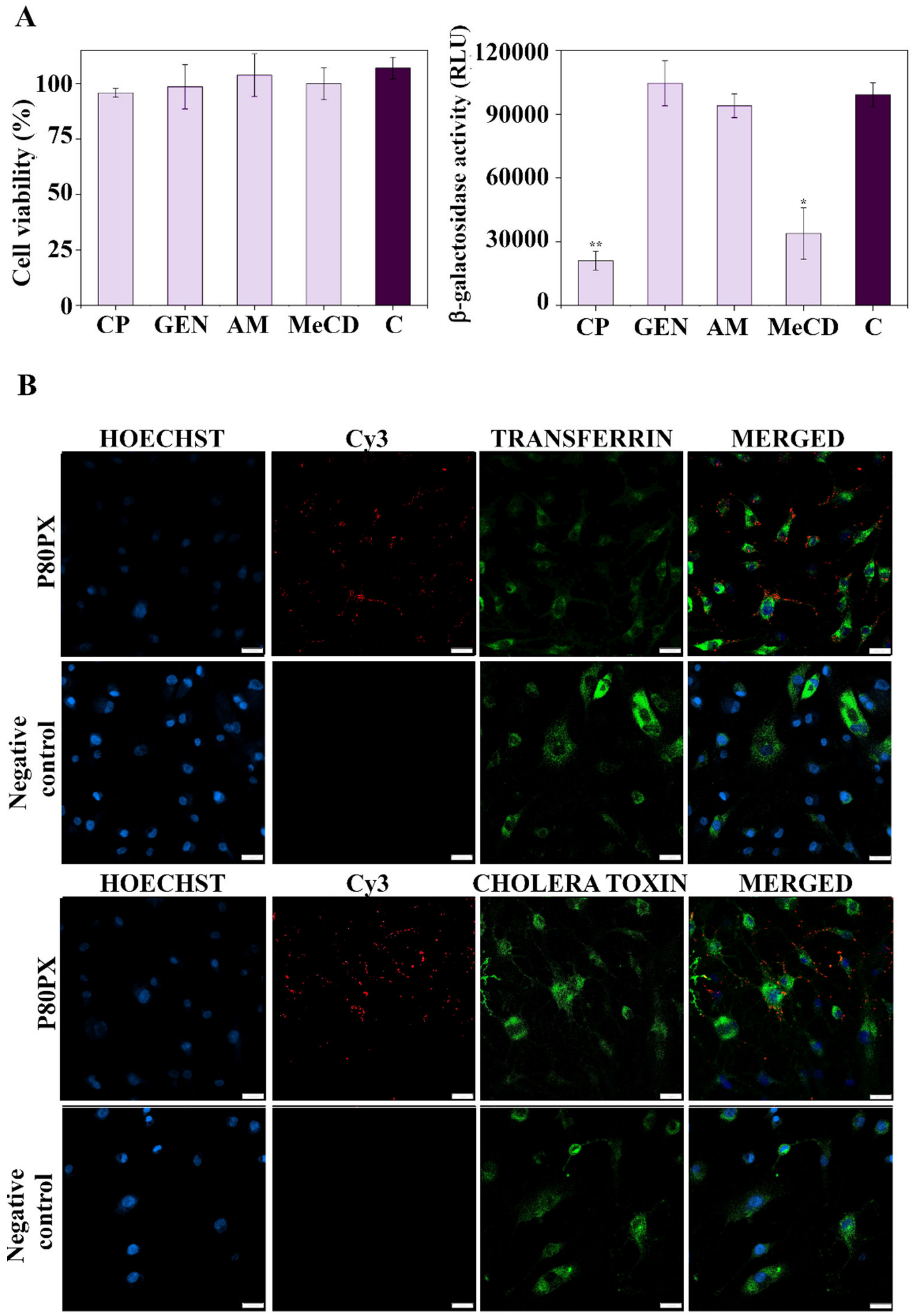

Figure S4. A. Cell viability (left) and β-galactosidase activity (right) of MSCs pre-treated or not (C: control) with endocytosis inhibitors: CP; chlorpromazine; GEN; genistein; AM; amiloride and MeCD; methyl-β-cyclodextrin) and subsequent transfection with P80PX nioplexes (10/1). **B.** Confocal microscopy representative images (magnification 10x scale bar 100 µm) showing the intracellular distribution of P80PX nioplexes (10/1) in MSCs. Blue coloring shows cell nuclei stained with Hoechst 33342, red color shows Cy3-labeled-p*lacZ* nioplexes and green coloring shows cells stained with AlexaFluor488-Transferrin or AlexaFluor488-Cholera toxin.

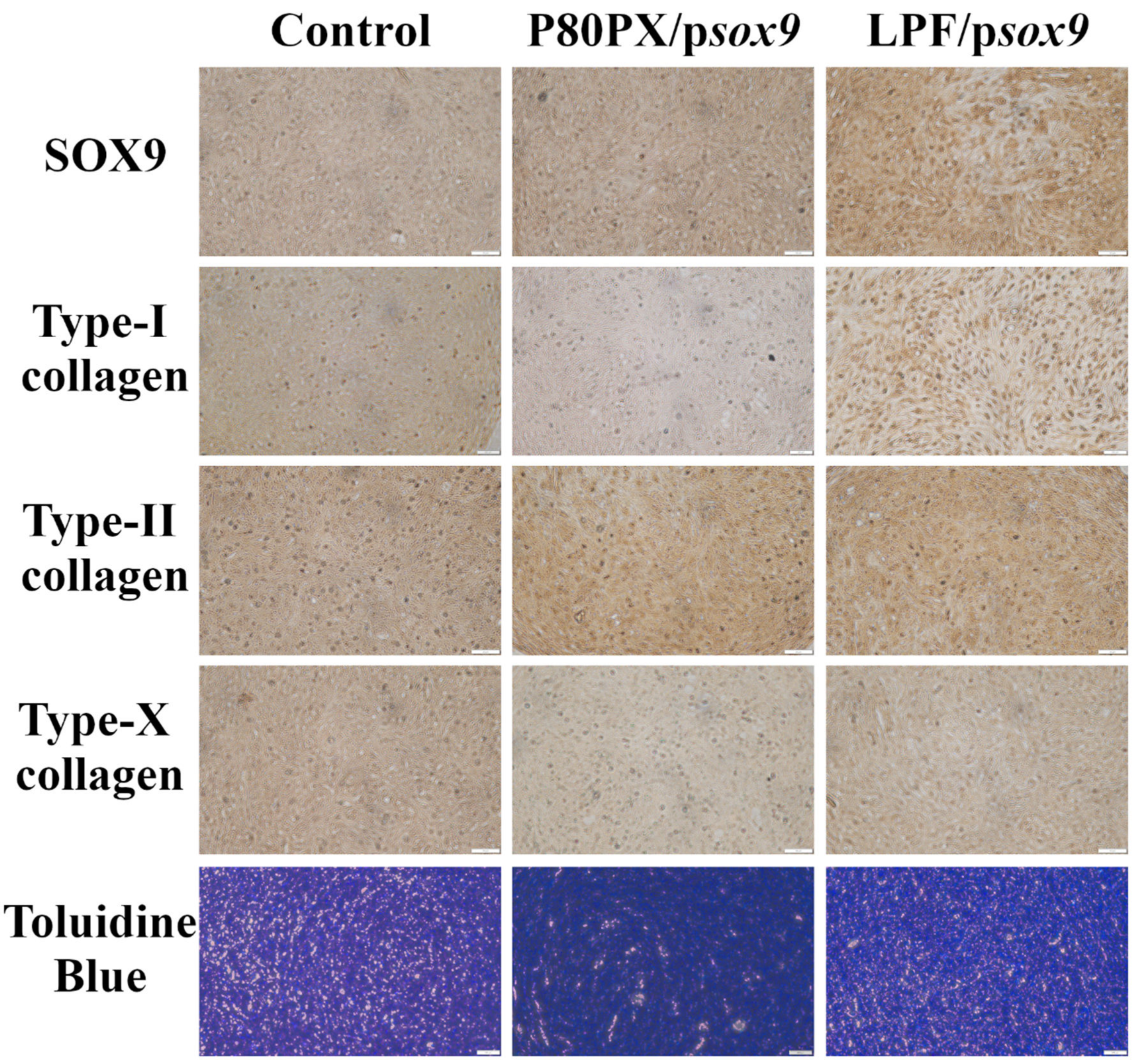

Figure S5. Representative images (magnification 20X; scale bar 50 µm) used in the histomorphometrical analysis of hMSCs aggregates cultured in chondrogenic medium (control; negative control), and transfected with psox9 via P80PX (P80PX/psox9) or LPF (LPF/psox9). Samples were kept in culture for 21 days and processed for immunodetection of SOX9, type-I, type-II and type-X collagen and toluidine blue.
